# Computational Model of Steroidogenesis in Human H295R Cells to Predict Biochemical Response to Endocrine-Active Chemicals: Model Development for Metyrapone

**DOI:** 10.1289/ehp.0901107

**Published:** 2009-10-16

**Authors:** Michael S. Breen, Miyuki Breen, Natsuko Terasaki, Makoto Yamazaki, Rory B. Conolly

**Affiliations:** 1 National Exposure Research Laboratory; 2 National Center for Computational Toxicology, U.S. Environmental Protection Agency, Research Triangle Park, North Carolina, USA; 3 Biomathematics Program, Department of Statistics, North Carolina State University, Raleigh, North Carolina, USA; 4 Safety Research Laboratory, Mitsubishi Tanabe Pharma Corporation, Kisarazu, Chiba, Japan

**Keywords:** endocrine-disrupting chemicals, H295R cells, mathematical model, mechanistic computational model, metyrapone, sensitivity analysis, steroid biosynthesis

## Abstract

Background: An *in vitro* steroidogenesis assay using the human adrenocortical carcinoma cell line H295R is being evaluated as a possible screening assay to detect and assess the impact of endocrine-active chemicals (EACs) capable of altering steroid biosynthesis. Data interpretation and their quantitative use in human and ecological risk assessments can be enhanced with mechanistic computational models to help define mechanisms of action and improve understanding of intracellular concentrationresponse behavior.

Objectives: The goal of this study was to develop a mechanistic computational model of the metabolic network of adrenal steroidogenesis to estimate the synthesis and secretion of adrenal steroids in human H295R cells and their biochemical response to steroidogenesis-disrupting EAC.

Methods: We developed a deterministic model that describes the biosynthetic pathways for the conversion of cholesterol to adrenal steroids and the kinetics for enzyme inhibition by metryrapone (MET), a model EAC. Using a nonlinear parameter estimation method, the model was fitted to the measurements from an *in vitro* steroidogenesis assay using H295R cells.

Results: Model-predicted steroid concentrations in cells and culture medium corresponded well to the time-course measurements from control and MET-exposed cells. A sensitivity analysis indicated the parameter uncertainties and identified transport and metabolic processes that most influenced the concentrations of primary adrenal steroids, aldosterone and cortisol.

Conclusions: Our study demonstrates the feasibility of using a computational model of steroidogenesis to estimate steroid concentrations *in vitro*. This capability could be useful to help define mechanisms of action for poorly characterized chemicals and mixtures in support of predictive hazard and risk assessments with EACs.

## Editor's Summary

*In vitro* systems to identify and characterize effects of endocrine-active chemicals (EACs) may reduce screening costs, testing time, and animal use while providing an experimental platform to characterize molecular and biochemical effects of exposure. The human adrenocortical carcinoma cell line H295R expresses all key enzymes involved in the synthesis of adrenocorticol steroid hormones from cholesterol and is being evaluated as a potential screening assay for EACs with effects on steroid biosynthesis. Breen et al. (p. 265) developed a mechanistic computational model to estimate the synthesis and secretion of 14 adrenal steroids in H295R cells and biochemical responses to enzyme inhibition by the model EAC metryrapone (MET), and they compared model estimates with time-course measurements in control and MET-exposed H295R cells. The authors report that predictions corresponded well with observed effects overall and conclude that results support the utility of this approach. They suggest that additional model development can facilitate research on unknown mechanisms of action and effects of chemical mixtures, and may improve the accuracy of low-dose extrapolations for risk assessment.

There is international concern about the potential for various environmental contaminants and commercial products to alter endocrine system function and contribute to adverse effects in humans and wildlife (Cooper and Kavlock 1997; Daston et al. 2003; Hutchinson et al. 2006; Zacharewski 1998). The Safe Drinking Water Act Amendments (1996) and the Food Quality Protection Act (1996) require screening for endocrine-disrupting properties of chemicals in drinking water and pesticides used in food production. In response to this legislation, the U.S. Environmental Protection Agency developed and implemented an endocrine disruptor screening program. The effort focuses on the effects of chemicals that mimic hormones by acting as agonists or antagonists of estrogen and androgen hormone receptors (Chu et al. 2009; Henley and Korach 2006), and other endocrine-active chemicals (EACs) that can cause effects by non-receptor-mediated mechanisms (Harvey and Everett 2003; Ulleras et al. 2008; Villeneuve et al. 2007). In this article, we describe a mechanistic computational model of steroidogenesis that can be used to estimate the biochemical effect of EACs that can modulate the activity of steroidogenic enzymes and the subsequent concentrations of steroid hormones.

Steroids have an important role in several physiologic and pathologic processes, such as stress response, development, metabolism, electrolyte regulation, reproduction, and hormone-sensitive cancers (Portier 2002; Ulleras et al. 2008). Steroids are derived from cholesterol (CHOL) and are synthesized primarily in the adrenal cortex, ovaries, testes, and placenta through a series of biochemical reactions mediated by multiple cytochrome P450 (CYP) enzymes and hydroxysteroid dehydrogenases (HSDs) (Miller 1988; Payne and Hales 2004). Exposure to various environmental EACs can alter the activity of these steroidogenic enzymes and the subsequent production rate of steroids (Sanderson 2006; Sanderson et al. 2002; Walsh et al. 2000). To better understand the intracellular mechanisms underlying the concentrationresponse behavior of steroidogenesis-disrupting chemicals, we are developing mechanistic computational steroidogenesis models that describe chemical-mediated biological perturbations at the biochemical level.

Data for our computational model were obtained from an *in vitro* steroidogenesis assay using the human adrenocortical carcinoma cell line H295R. The H295R cells express all the key enzymes for steroidogenesis and the ability to produce all the adrenocorticol steroids (Gazdar et al. 1990; Rainey et al. 1994; Staels et al. 1993). The expression of steroidogenic genes in H295R cells is well correlated to the expression in normal human adrenal (Oskarsson et al. 2006). The H295R cell line has been widely used to study adrenocortical function, regulation of steroidogenesis, and screening of EACs (Gracia et al. 2006; Hecker and Giesy 2008; Muller-Vieira et al. 2005; Sanderson et al. 2002; Ulleras et al. 2008). The H295R assay system is being developed and evaluated by several international laboratories as a possible steroidogenesis screening approach (Hecker et al. 2007). This assay coupled with a mechanistic computational model supports the recommendations by the National Research Council (2007) on the vision of toxicology in the 21st century with the use of *in vitro* systems that can *a*) provide broad coverage of chemicals, mixtures, and outcomes; *b*) reduce the cost and time of testing; *c*) use fewer animals; and *d*) develop a more robust scientific basis to assess health effects from environmental agents.

A mechanistic mathematical model of steroidogenesis has several potential applications. First, this type of model can enhance the interpretation of data from *in vitro* steroidogenesis assays by helping to define mechanisms of action for poorly characterized chemicals and mixtures of chemicals in support of *in vitro* EAC screening methods. Second, this model can help guide low-concentration extrapolations of *in vitro* concentrationresponse curves. Third, the model can help formulate hypotheses and design critical experiments. Fourth, a model that predicts the response of the major adrenal steroids [e.g., cortisol (CORT), aldosterone (ALDO)] to EACs can be coupled to multiorgan systems models, which include regulatory feedback of the hypothalamuspituitaryadrenal axis and the renalangiotensinaldosterone system, in support of *in vivo* EAC screening methods.

Other steroidogenesis models have been previously reported. Murphy et al. (2005) developed a model for vitellogenesis, a steroid-controlled process, in female fish. To model ovarian steroidogenesis, all reactions between the release of gonadotropin and the production of testosterone were combined and mathematically described by one Hill equation. Selgrade and Schlosser (1999) developed a mathematical model to predict plasma levels of estradiol during different stages of the menstrual cycle in women. Estradiol concentrations were modeled as a weighted sum of luteinizing hormone, growth follicle stage, and preovulatory stage. However, these models lack a mechanistic metabolic pathway of steroid biosynthesis at the biochemical level. Breen et al. (2007) developed a mechanistic computational model of ovarian steroidogenesis. Metabolic reaction and transport rates were estimated from ovary explants of a small fish. Becker et al. (1980) developed a probabilistic model of the metabolic pathway for testicular steroidogenesis. Transition probabilities for the reactions in the pathway were estimated from *ex vivo* preparations of rat and rabbit testes. However, ovarian and testicular steroidogenesis does not include the metabolic pathways for the major adrenal steroids, aldosterone and cortisol.

In this study, we developed a mechanistic computational model of the adrenal metabolic and transport processes that mediate steroid synthesis and secretion and the kinetics for enzyme inhibition by the competitive steroidogenic enzyme inhibitor metyrapone (MET), a model EAC.

## Materials and Methods

We first describe the *in vitro* steroidogenesis experiments, and then the mathematical model and procedures for parameter estimation.

*Steroidogenesis assay with H295R cells.* We performed two experimental studies with H295R cells: a control study with samples analyzed at five time points (0, 8, 24, 48, and 72 hr) and a MET study with two MET concentrations (1 and 10 µM) with samples analyzed at four time points [8, 24, 48, and 72 hr; see Supplemental Material for details (doi:10.1289/ehp-118-265.S1 via http://dx.doi.org/)]. Briefly, the medium and cells were separately removed from four replicate wells at each time point. The cells were dissolved in 100 µL distilled water and sonicated to produce a cell lysate. Steroid concentrations in the medium and cell lysate were measured using liquid chromatography/mass spectrometry for 12 steroids [pregnenolone (PREG), 17〈-hydroxypregnenolone (HPREG), dehydroepiandrosterone (DHEA), progesterone (PROG), 17〈-hydroxyprogesterone (HPROG), androstenedione (DIONE), testosterone (T), deoxycorticosterone (DCORTICO), corticosterone (CORTICO), ALDO, 11-deoxycortisol (DCORT), and CORT] and using enzyme-linked immunosorbent assay for two additional steroids [estrone (E_1_) and 17®-estradiol (E_2_)]. The quantitative ranges for each steroid in the cells and medium are provided in Table 1 of the Supplemental Material (doi:10.1289/ehp-118-265.S1).

**Table 1:** Estimated transport equilibrium parameter values (dimensionless) and *R^2^* values from model fit of steroids corresponding to given *q* parameters.

Parameter	Value	*R^2^*
*q*_19_	0.0048	0.98
*q*_20_	0.0019	0.97
*q*_21_	0.0140	0.99
*q*_22_	0.0171	0.99
*q*_23_	0.0268	0.98
*q*_24_	0.0229	0.97
*q*_25_	0.0072	0.99
*q*_26_	0.0141	0.97
*q*_27_	0.0201	0.99
*q*_28_	0.0174	0.70
*q*_29_	0.0124	0.99
*q*_30_	0.0084	0.98
*q*_31_	0.0130	0.98
*q*_32_	0.0108	0.99
*q*_40_	0.0171	———^*a*^

*Estimation of cell volume.* To estimate the volume of the cells per well, we performed a cell morphology study following the same experimental method as the previously described steroidogenesis assay for both controls and the two concentrations of MET (1 and 10 µM). At post-stimuli incubation periods of 0, 24, 48, and 72 hr, cells were separated from the medium and removed from six replicate wells. The mean cell diameter and mean cell circularity in each well were measured using a cell analyzer (Vi-CELL XR, Beckman Coulter, Fullerton, CA, USA). Because the mean circularity of the separated cells was always ≥ 90%, a spherical cell shape was assumed with a volume *V*_indiv_cell_ expressed as


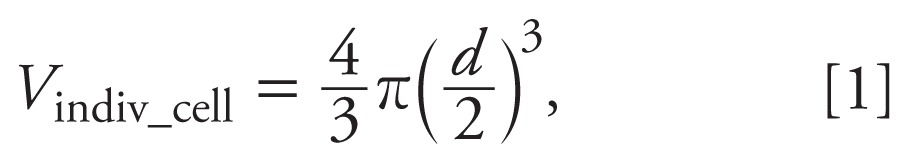


where *d* is the mean measured cell diameter (14.20 µm). This yielded a *V*_indiv_cell_ of 1,499 µm^3^. To determine the mean volume of cells per well, *V*_cell_, we multiplied *V*_indiv_cell_ by the number of cells per well.

*Compensation of steroid dilution in cell lysate.* To compensate for dilution of the steroids by 0.1 mL distilled water, *V*_water_, added to the cell lysate, we determined the concentration of steroid *x* in cells, *C*_cell,_*_x_*(*t*), by multiplying the measured concentration of steroid *x* in the cell lysate, *C*_lysate,_*_x_*(*t*), by the dilution factor *V*_lysate_/*V*_cell_, where the volume of the cell lysate, *V*_lysate_, is the sum of *V*_cell_ and *V*_water_.

**Figure 1:**
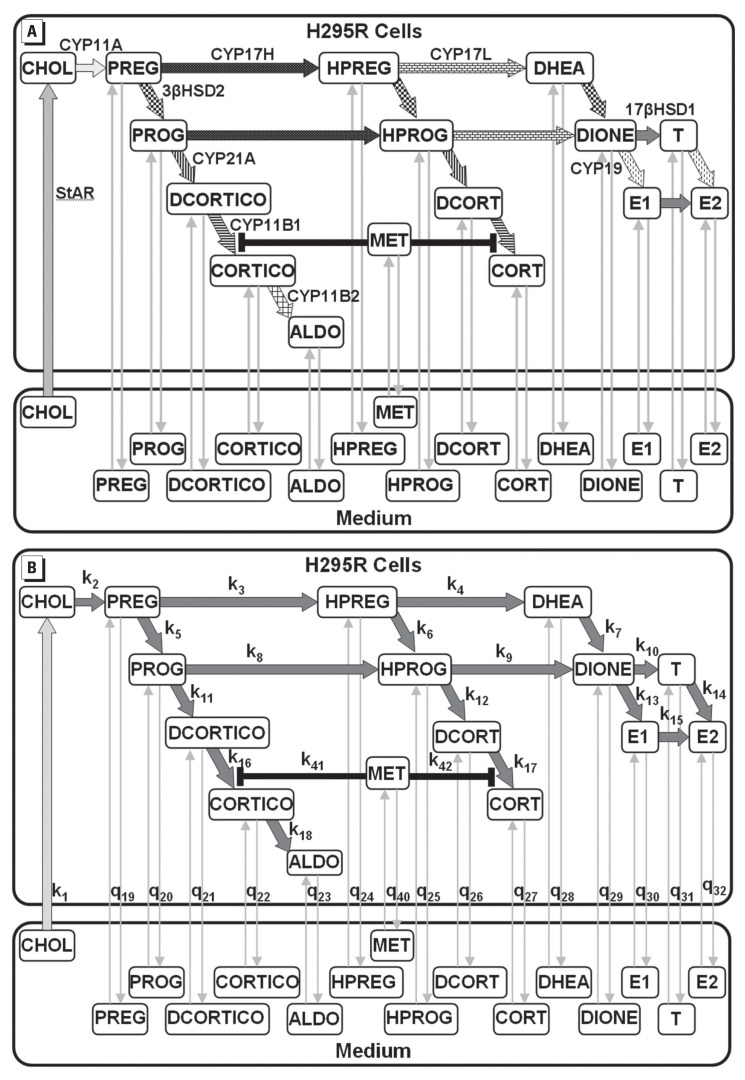
(*A*) Conceptual steroidogenesis model for control and MET‑exposed H295R cells. The model consists of two compartments: culture medium and H295R cells. Cellular uptake of CHOL from medium is depicted by the broad gray arrow labeled with the StAR protein. Reversible steroid transport between medium and cells is depicted by bidirectional thin gray arrows. Irreversible metabolic reactions in the cells are depicted by arrows, with each pattern representing a unique enzyme. Enzymes are labeled next to reactions they catalyze: CYP450 side‑chain‑cleavage (CYP11A), CYP450c17‑α‑hydroxylase (CYP17H), CYP450c17,20‑lyase (CYP17L), 3‑β‑hydroxydehydrogenase type 2 (3βHSD2), 17β‑hydroxydehydrogenase type 1 (17βHSD1), CYP450 aromatase (CYP19), CYP450 21-α-hydroxylase (CYP21A), CYP450 11‑β‑hydroxylase type 1 (CYP11B1), and aldosterone synthase (CYP11B2). Steroids are PREG, HPREG, DHEA, PROG, HPROG, DIONE, T, E_1_, E_2_, DCORTICO, CORTICO, ALDO, DCORT, and CORT. The EAC MET is shown as enzyme inhibitor of CYP11B1. (*B*) A graphical representation of the parameters for the mathematical H295R steroidogenesis model, which consists of first‑order rate constants for CHOL uptake into the cells, *k*_1_, and for each metabolic process, *k*_2_–*k*_18_. For the quasi‑equilibrium analysis, the equilibrium constants are *q*_19_–*q*_32_. Partition coefficient for MET is *q*_40_. Enzyme inhibition constants for MET are *k*_41_ and *k*_42_ for CORTICO and CORT pathways, respectively.

*Overview of mathematical H295R steroidogenesis model.* The computational model is based on an *in vitro* steroidogenesis experimental design with two compartments: culture medium and H295R cells (Figure 1). The model consists of steroid transport and metabolic pathways. The transport pathways include cellular uptake of CHOL (steroid precursor) and MET and the import and secretion of 14 adrenal steroids (PREG, HPREG, DHEA, PROG, HPROG, DIONE, T, DCORTICO, CORTICO, ALDO, DCORT, CORT, E_1_, and E_2_). The metabolic pathway includes conversion of CHOL into the 14 adrenal steroids and inhibition of steroidogenic enzymes by MET. Development of various aspects of the model is described in detail below.

*Import of CHOL, the precursor for all steroid hormones.* Cholesterol is transported to the inner mitochondrial membrane, which is the site for the first metabolic reaction of steroid biosynthesis. This transport process consists of two main steps. First, CHOL is imported into the cell mainly by the low-densityliproprotein-receptormediated lysosomal pathway (Brown and Goldstein 1986; Chang et al. 2006; Gallegos et al. 2000). Second, CHOL is delivered to the inner mitochondrial membrane by the intracellular sterol carrier protein-2, steroidogenic acute regulatory (StAR) protein, and peripheral benzodiazepine receptor (Chang et al. 2006; Gallegos et al. 2000; Maxfield and Wustner 2002). We model the transport rate of CHOL from the medium as a first-order process (Figure 1B).

*Metabolic pathway.* The metabolic pathway in the H295R cells that converts CHOL into the 14 adrenal steroids consists of 17 enzymatic reactions catalyzed by nine different proteins (Figure 1A) (Payne and Hales 2004). All metabolic reactions occur in the smooth endoplasmic reticulum except conversion of CHOL to PREG, which occurs in the inner mitochondrial membrane (Agarwal and Auchus 2005; Miller and Strauss 1999). Interorganelle transports are not included in the model because we assumed these processes are not rate limiting. Because the metabolic reactions are predominantly irreversible, the reverse reaction rates are set to zero (Becker et al. 1980). We assume the substrate concentration is much less than the Michaelis constant (substrate concentration that yields a half-maximal reaction rate). Thus, the rate of product formation increases linearly with substrate concentration as described by a first-order rate constant (Figure 1B).

*Steroid transport pathway.* The transport of the steroids between the cells and medium is mediated by multiple transport mechanisms, including nonvesicular and vesicular processes (Chang et al. 2006; Maxfield and Wustner 2002; Neufeld et al. 1996). Because the concentration of the newly synthesized steroids in the cells is probably insufficient to saturate the multiple steroid transport mechanisms during the experiments, we model the rates of secretion and uptake for each steroid as reversible first-order processes [*k*_+_*_x_* and *k_x_* for secretion and uptake of steroid *x*, respectively; see Supplemental Material, Figure 1 (doi:10.1289/ehp-118-265.S1)].

*Uptake and enzyme inhibition by MET.* Various EACs can directly inhibit the steroidogenic enzymes in the metabolic pathway. In this study, we examined the steroid response of H295R cells to exposures from MET, an EAC that is a competitive inhibitor of CYP11-®-hydroxylase (CYP11B1), which catalyzes two different reactions in the metabolic pathway: conversion of DCORTICO to CORTICO, and conversion of DCORT to CORT (Figure 1A) (Harvey and Everett 2003; Harvey et al. 2007). We assume that MET diffuses into the cells and reaches equilibrium with the MET concentration in the medium:

*C*_MET,cell_(*t*)=*q*_40_*C*_MET, med_(*t*), [2]

where *C*_MET,cell_ and *C*_MET,med_ are the cell and medium MET concentrations at time *t*, respectively, and *q*_40_ is the partition coefficient (Figure 1B). To account for the volumes of the cells, Vcell, and medium, Vmed, the molecular balance equation

*V*_cell_*C*_MET,cell_(*t*)+*V*_med_*C*_MET,med_(*t*) =*V*_cell_*C*_MET,cell_(0)+*V*_med_*C*_MET,med_(0), [3]

is solved for *C*_MET,med_(*t*) and substituted into Equation 2 with *C*_MET,cell_(0) = 0 to yield


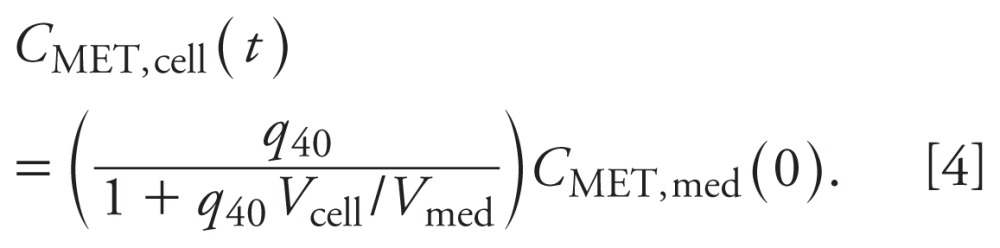


For the two CYP11B1 enzymatic reactions competitively inhibited by MET, the kinetic parameters *k*_16_ and *k*_17_ are respectively divided by 〈_CORTICO_ = 1 + (*C*_MET,cell_/*k*_41_) and 〈_CORT_ = 1 + (*C*_MET,cell_/*k*_42_) with MET inhibition constants *k*_41_ and *k*_42_ (Figure 1B).

*Dynamic molecular balances.* The time courses of the steroids are described by dynamic molecular balance equations [see Supplemental Material (doi:10.1289/ehp-118-265.S1)]. The dynamic molecular balance equations for the steroids in cells and medium are


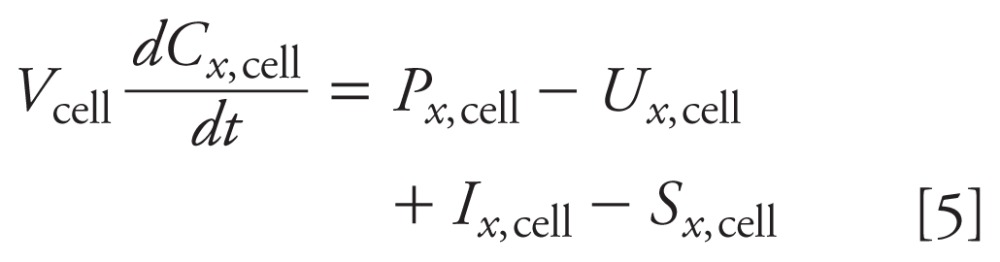


and


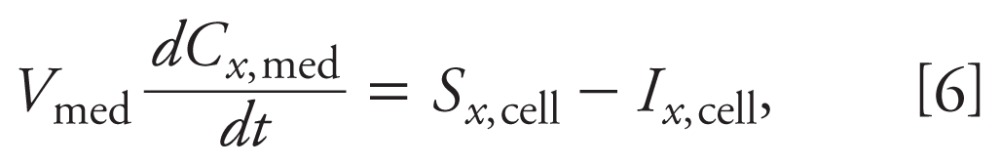


where *C_x_*_,cell_ and *C_x_*_,med_ are the concentrations of steroid *x* in cells and medium, respectively; *P_x_*_,cell_ and *U_x_*_,cell_ are the production and use rates of steroid *x* in cells, respectively; *I_x_*_,cell_ and *S_x_*_,cell_ are the cell import and secretion rates of steroid *x*, respectively. The first two terms on the right side of Equation 5 represent the net metabolic reaction rate of steroid *x*. The last two terms represent the net cellular uptake or release rate of steroid *x*.

*Quasi-equilibrium analysis.* We assume that the steroid concentrations in the cells and medium are operating near equilibrium. There is good experimental evidence to support this assumption. First, the time-course data from the control and MET-exposed cells show that some steroid concentrations in the medium increase for 48 hr but then decrease at 72 hr. Because the cells can secrete and import steroids, the steroid transport is probably reversible. Second, the time-course data from the control and MET studies show remarkably similar dynamic behavior for each steroid concentration in the cells and its corresponding concentration in medium. For each steroid, a comparison between the simultaneous measurements in the cells and medium shows that a linear regression line (y-intercept set to zero) closely fits the data [see Supplemental Material, Figure 2 (doi:10.1289/ehp-118-265.S1)]. This linear correlation between concentrations in the cells and medium is clearly evident with large *R*^2^ values for each steroid transport parameter (Table 1). This is good evidence that the steroid transport between the cells and medium is rapid and reversible. Therefore, we assume that the steroid concentrations in the cells and medium reach equilibrium after a short transient time. Because the steroids are also involved in the metabolic pathway of steroidogenesis, this is considered a quasi-equilibrium.

**Figure 2:**
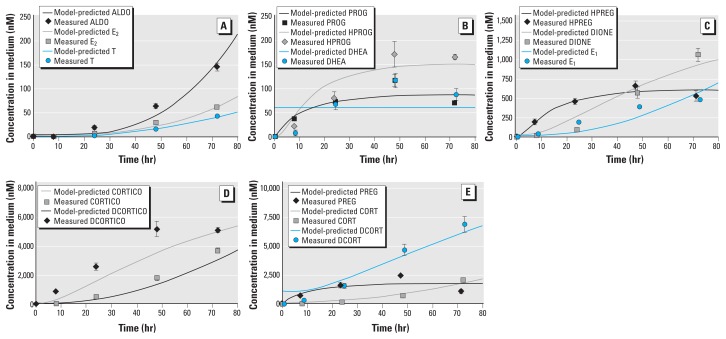
Model evaluation of metabolic and transport pathways for control study. Model-predicted concentrations in medium were plotted as a function of time and compared with concentrations (mean ± SD) measured at five sampling times for steroids: ALDO, E_2_, and T (*A*); PROG, HPROG, and DHEA (*B*); HPREG, DIONE, and E_1_ (*C*); CORTICO and DCORTICO (*D*); and PREG, CORT, and DCORT (*E*).

To examine the quasi-equilibrium behavior, the reversible transport rates (*k*_+_*_x_* and *k_x_* for secretion and import of steroid *x*, respectively) are assumed to be much faster than the metabolic reaction rates. After a short period of time, the concentration of steroid *x* in the cells and medium reaches equilibrium:


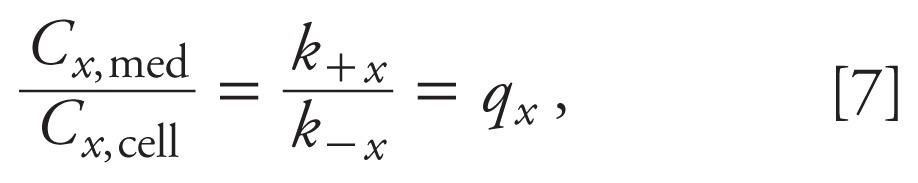


where *q_x_* is the equilibrium constant. We can sum the mass (molecules) of steroid *x* in the cells and medium to yield


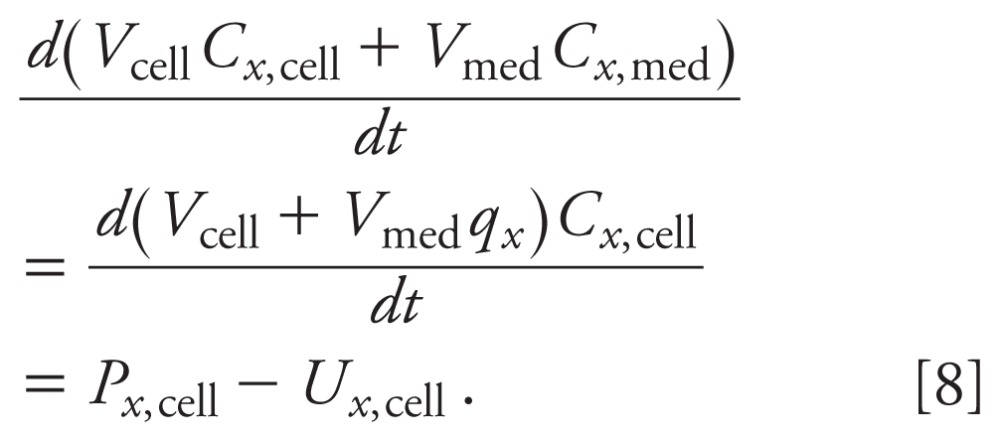


The simplified system of equations consists of a differential equation for each steroid in the cells,


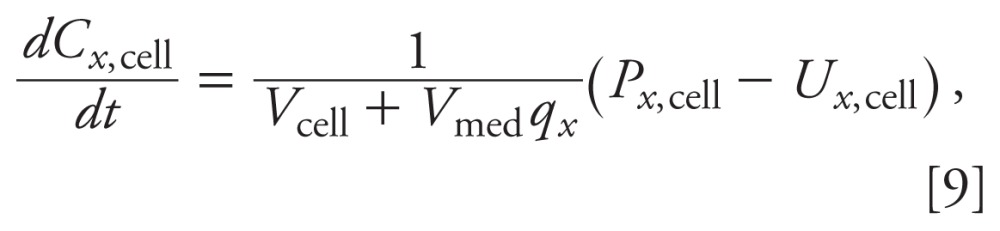


and an algebraic equation for each steroid in the medium,

*C_x_*_,med_=*q_x_C_x_*_,cell_. [10]

The model consists of 14 transport equilibrium constants (*q*_19_, *q*_20_, . . ., *q*_32_), 17 metabolic rate constants (*k*_2_, *k*_3_, . . ., *k*_18_), a CHOL import rate (*k*_1_), two enzyme inhibition constants for MET (*k*_41_, *k*_42_), and the partition coefficient for MET (*q*_40_). These dynamic molecular balance equations for quasi-equilibrium and 35 parameters are used in all subsequent analyses [see Supplemental Material (doi:10.1289/ehp-118-265.S1)].

*Parameter estimation.* The parameters for the two pathways (steroid transport pathway and metabolic pathway) were independently estimated using the mean concentrations from replicate experiments. For the steroid transport pathway, the equilibrium constants (*q*_19_, *q*_20_, . . ., *q*_32_), were estimated with the time-course data from the control and MET studies using the direct least squares solution for Equation 10:

q*_x_=_[_*C_x_*_,cell_´ *C_x_*_,cell__]_^-1^*C_x_*_,cell_´ *C_x_*_,med_, [11]

where q_x_* is the least squares estimate of the equilibrium constant for steroid *x*, and *C_x_*_,cell_´ = [*C_x_*_,cell_(*t* = 0, *d* = 0) *C_x_*_,cell_(*t* = 8, *d* = 0) . . . *C_x_*_,cell_(*t* = 72, *d* = 10)] and*C_x_*_,med_´ = [*C_x_*_,med_(*t* = 0, *d* = 0) *C_x_*_,med_(*t* = 8, *d* = 0) . . . *C_x_*_,med_(*t* = 72, *d* = 10)] are the measured concentrations in the cell and medium, respectively, at time *t* for the MET dose *d* for *d* = 0, 1, and 10 µM.

For the metabolic pathway, the parameters (*k*_1_, *k*_2_, . . ., *k*_18_, *k*_41_, *k*_42_) were estimated with the time-course data from the control and MET studies using the weighted least squares method. Let *C_x_*_,cell_(*t_i_*; *C^d^*_MET,med_, *k*) be the model-predicted concentrations of steroid *x* in the cells at the *i*th time *t_i_* for the *d*th MET dose (including control) *C^d^*_MET,med_ with parameter set *k* = (*k*_1_, *k*_2_, . . ., *k*_18_, *k*_41_, *k*_42_). Let *C^d^*^,^*^i^_x_*_,cell_ be the measured concentration of steroid *x* in the cells at the *i*th time *t_i_* for the *d*th MET dose (including control) *C^d^*_MET,med_, and let *C^d^_x_*_,cell_ be the mean measured concentration across time where *d* = 1, . . ., 3 and *i* = 1, . . ., 5. Then, the weighted least squares estimate, k* = (*k*_1_*, *k*_2_*, . . ., *k*_18_*, *k*_41_*, *k*_42_*), is the parameter values *k*, which minimizes the cost function


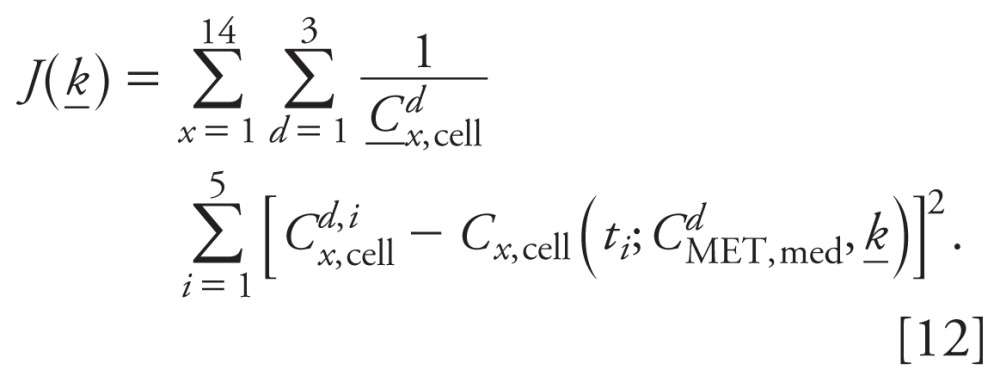


Parameters for the metabolic pathway were estimated with an iterative optimization algorithm using MATLAB R2009a (Mathworks, Natick, MA, USA) software. We chose the Nelder-Mead simplex method for its relative insensitivity to the initial parameter values compared with other common methods, such as Newton’s method, and its robustness to discontinuities (Nelder and Mead 1965). Convergence to the solution was confirmed after the parameter search terminated.

*Sensitivity analysis.* We performed a sensitivity analysis to examine model uncertainty. The sensitivity function relates the changes of the model output to changes in the model parameters. To rank the sensitivity functions, we calculated relative sensitivity functions *R_x_*_,med,_*_ki_* with respect to parameter *k_i_* for each of the model-predicted concentrations in the medium *C_x_*_,med_ as described by


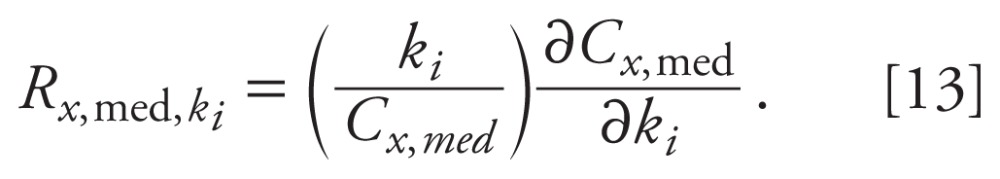


Substituting Equation 10 into Equation 13 yields


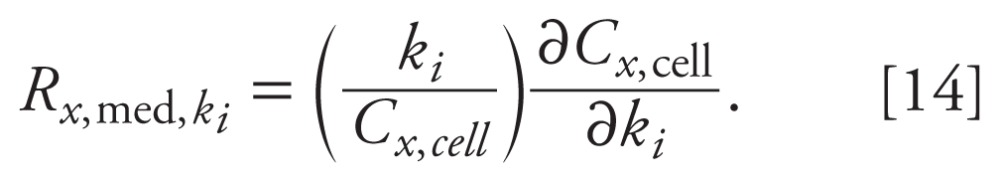


The relative sensitivities *R_x_*_,med,_*_qi_* with respect to parameter *q_i_* for each of the model-predicted concentrations in the medium *C_x_*_,med_ are


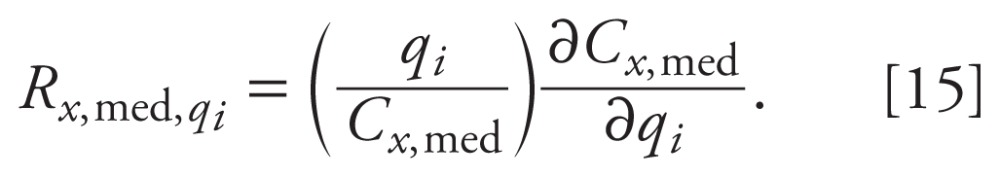


Substituting Equation 10 into Equation 15 yields


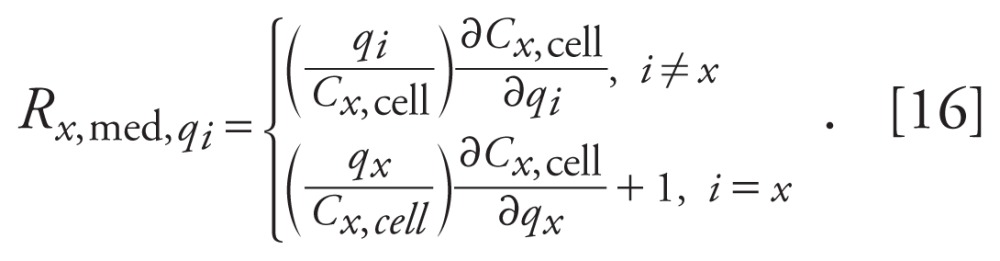


Using MATLAB, partial derivatives were numerically determined for *C_x_*_,cell_ with respect to each parameter, and relative sensitivity functions were calculated as shown in Equations 14 and 16 for control and each MET dose. To rank the relative sensitivities, we calculated the L2 norm across time for each relativity sensitivity function as described by


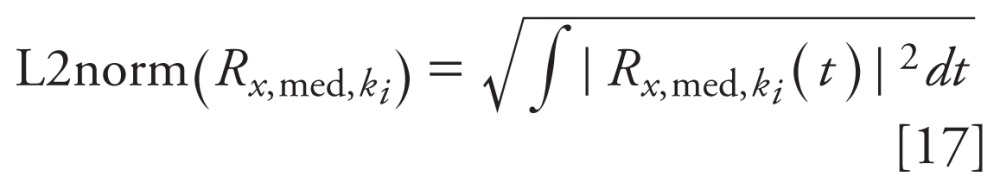


and


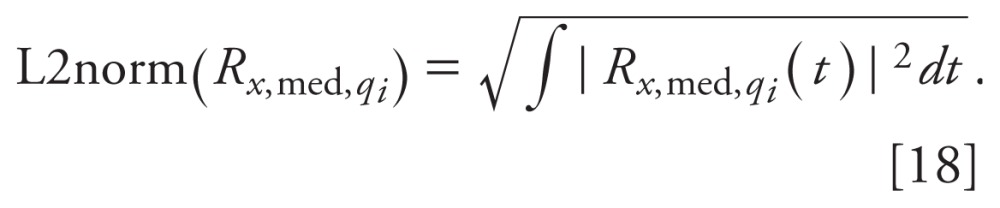


Magnitudes of the relative sensitivities relate the degree to which changes in parameters values lead to changes in model outputs.

## Results

*Transport pathway.* Table 1 shows the estimated parameter values and *R*^2^ values for the model evaluation of the transport pathway. The MET transport equilibrium (*q*_40_) could not be determined from the data because MET was not measured in the cells. Therefore, we set (*q*_40_) equal to CORTICO transport equilibrium (*q*_22_) because the previously measured partition coefficients for MET (*X*log*P* = 2.0) and CORTICO (*X*log*P* = 1.9) are similar [PubChem Database (National Center for Biotechnology Information 2003)]. The transport equilibrium model predictions correspond well to the mean steroid concentrations measured in the cells and medium with large *R*^2^ values (Table 1). For DCORTICO, the transport equilibrium model closely fits the measured concentrations in the cells and medium [see Supplemental Material, Figure 2 (doi:10.1289/ehp-118-265.S1)]. Across time, the model-predicted and measured DCORTICO concentrations in medium also correspond well [see Supplemental Material, Figure 3 (doi:10.1289/ehp-118-265.S1)]. Similar results were observed for the other steroids. The close fit of a transport equilibrium model to the data indicates that the steroid concentrations in the cells and medium reach equilibrium after a short time.

**Figure 3:**
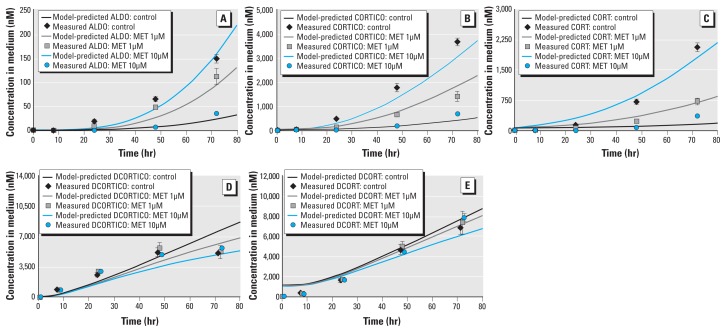
Model evaluation of metabolic and transport pathways for control and two MET concentrations (1 µM and 10 µM). Model‑predicted concentrations in medium were plotted as a function of time and compared with concentrations (mean ± SD) measured at five sampling times for steroids: ALDO (*A*), CORTICO (*B*), CORT (*C*), DCORTICO (*D*), and DCORT (*E*). For controls, model‑predicted and measured steroid concentrations are the same as shown in Figure 2.

*Metabolic pathway.* Table 2 shows the estimated parameter values for the metabolic pathway. The time for convergence to the solution for the iterative parameter estimation was typically 24 min on an Intel Core 2 Duo processor computer using MATLAB.

**Table 2:** Estimated parameter values of metabolic pathway.

Parameter	Value	Unit
*k*_1_	0.0049	hr^-1^
*k*_2_	0.0230	hr^-1^
*k*_3_	0.9448	hr^-1^
*k*_4_	2.7 x 10^-9^	hr^-1^
*k*_5_	0.8522	hr^-1^
*k*_6_	13.2263	hr^-1^
*k*_7_	0.0020	hr^-1^
*k*_8_	3.1 x 10^-5^	hr^-1^
*k*_9_	3.1479	hr^-1^
*k*_10_	0.0367	hr^-1^
*k*_11_	6.8701	hr^-1^
*k*_12_	13.6062	hr^-1^
*k*_13_	0.5482	hr^-1^
*k*_14_	0.0003	hr^-1^
*k*_15_	0.0828	hr^-1^
*k*_16_	0.5627	hr^-1^
*k*_17_	0.2396	hr^-1^
*k*_18_	0.0847	hr^-1^
*k*_41_	18.1767	nM
*k*_42_	8.2661	nM

For control cells, we compared model- predicted steroid concentrations with time-course measurements. Overall, the model- predicted concentrations correspond well to the mean time-course data in cells [see Supplemental Material, Figure 4 (doi:10.1289/ehp-118-265.S1)] and in medium (Figure 2). For two steroids (PROG and PREG) with mean measurements that increase until 48 hr and then sharply decrease at 72 hr, the model underestimated at 48 hr and overestimated at 72 hr (Figure 2B,E). For DCORTICO, the model underestimated the mean measurements at 8, 24, and 48 hr (Figure 2D). For DHEA, all model-predicted and measured concentrations in the cells were below the minimum level of quantification [see Supplemental Material, Figure 4 (doi:10.1289/ehp-118-265.S1)]. Therefore, the ability of the model to accurately correspond to the time-varying concentrations of DHEA measured in the medium is limited with the assumed quasiequilibrium between the cells and medium. The model-predicted DHEA concentrations in the medium correspond well with the average time-course behavior of the measurements (Figure 2B).

**Figure 4:**
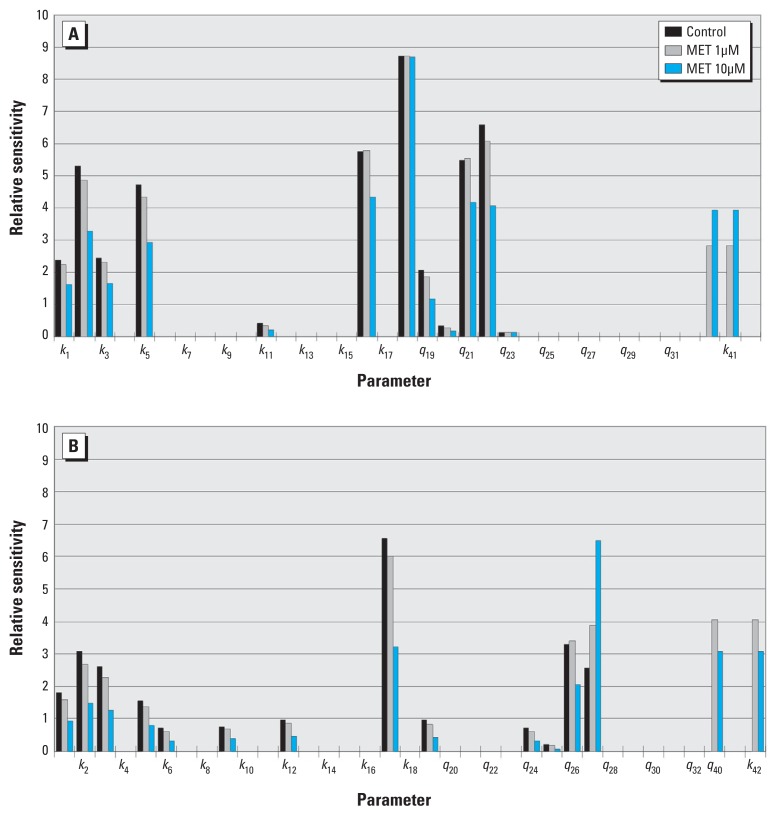
Relative sensitivities for model‑predicted steroids ALDO (*A*) and CORT (*B*), plotted as a function of the 35 model parameters (k_1_–k_18_, q_19_–q_32_, k_40_–k_42_) for control and two MET concentrations (1 and 10 µM). Each bar represents the L2 norm of the relative sensitivities across time (0–80 hr) and indicates the degree to which changes in parameter values lead to changes in model outputs. Odd- and even-numbered parameters are shown in *A* and *B*, respectively.

For MET-exposed cells, we compared model-predicted steroid concentrations with time-course measurements after incubation with MET. For the steroids (CORTICO, ALDO, and CORT) downstream from the enzyme inhibited by MET (CYP11B1), the model-predicted concentrations closely correspond to the mean time-course measurements in cells [see Supplemental Material, Figure 5 (doi:10.1289/ehp-118-265.S1)] and in medium (Figure 3AC), which decrease as MET increases. For the steroids (DCORTICO and DCORT) immediately upstream from CYP11B1, the model-predicted concentrations compare well with the mean time-course data in cells [see Supplemental Material (doi:10.1289/ehp-118-265.S1)] and in medium (Figure 3D,E), which remain approximately unchanged at 8, 24, and 48 hr as MET increases and then slightly increase at 72 hr as MET increases. For the other steroids further upstream from CYP11B1, the model-predicted and measured concentrations remained approximately unchanged from controls as MET increases (data not shown).

*Sensitivity analysis.* Figure 4 shows the relative sensitivities for two steroids. Odd- and even-numbered parameters are shown in Figure 4A and 4B, respectively. For ALDO, two parameters (*k*_18_, *q*_22_) were highly sensitive at each MET dose, and six parameters were moderately sensitive, with their sensitivity decreased (*k*_2_, *k*_5_, *k*_16_, *q*_21_) or increased (*k*_41_, *q*_40_) as MET increased. For CORT, two parameters (*k*_17_, *q*_27_) were highly sensitive at each MET dose, and five parameters (*k*_2_, *k*_3_, *k*_26_, *q*_40_, *q*_42_) were moderately sensitive with their sensitivity decreased as MET increased. The HPREG pathway appears to be the preferred pathway for CORT synthesis because CORT was more sensitive to the HPREG pathway (*k*_3_, *k*_6_) and less sensitive to the PROG pathway (*k*_5_, *k*_8_). The sensitivity of ALDO and CORT can indicate the uncertainty of the parameters. The parameters with high sensitivity tend to have less uncertainty compared with parameters with low sensitivity.

## Discussion

We developed a mechanistic mathematical model and estimated metabolic and transport parameters for adrenal steroidogenesis to estimate synthesis and transport of the steroids and their dynamic concentrationresponse to the EAC MET. In the H295R cells and medium, the model-predicted steroid concentrations closely correspond to the time-course data from control experiments and dynamic concentrationresponse data from experiments with MET-exposed cells. The quasi-equilibrium assumption reduced the complexity of the model while maintaining the model’s predictive ability.

*Advantages of mechanistic model.* The potential importance of the model is due to the use of mechanistic information at the biochemical level. Our mechanistic model includes each enzymatic reaction in the metabolic pathway. Under control conditions, the rate-limiting step is the transport of CHOL from the outer to inner mitochondrial membrane (Chang et al. 2006; Miller and Strauss 1999). For EAC-exposed cells, one or more steps in the pathway can become rate limiting, depending on the EAC concentration and enzyme inhibition strength. Some chemicals inhibit a single specific steroidogenic enzyme, whereas others inhibit multiple enzymes (Harvey et al. 2007). After further development, our model should increase insight into mechanisms of steroidogenic-active chemicals with unknown mechanisms of action and mixtures of chemicals. Furthermore, laboratory experiments are often performed with EACs at higher doses than typical human exposures because of the quantification limits of the assay. Low concentration extrapolations of concentrationresponse curves may be inaccurate if not guided by mechanistic models (Conolly and Lutz 2004).

The experiments used to fit and evaluate this model included time-course measurements of each adrenal steroid in both the cells and medium. In addition, the mechanism of action for MET, the EAC used in this study, was previously characterized as a potential CYP11B1 enzyme inhibitor. These “data-rich” experiments allow us to fit and evaluate the model for each steroid. After further refinement and evaluation of the model for other EAC with different mechanisms of action, the model could be then applied for rapid *in vitro* EAC screening methods, which measure only a few steroids. The refined model would help identify mechanisms of action for poorly characterized EAC and extrapolate concentrationresponse curves in support of human hazard and risk assessments.

The model assumption of quasi-equilibrium has several advantages. It reduces the number of model parameters and the number of differential equations in the mathematical model by replacing some of them with algebraic equations. Also, it decouples the system of equations for the metabolic and transport pathways to allow the set of parameters for each pathway to be independently estimated. Moreover, it reduces the complexity of the more general model while preserving its important features and facilitating model analysis.

In vitro *steroidogenesis assay.* As shown with *in vitro* data, H295R cells can provide the data needed for comparison with model predictions. H295R cell experiments eliminate the feedback of the hypothalamuspituitaryadrenal axis, which allows discrimination among different modes of action for EACs. This *in vitro* assay can identify direct effects at the molecular and biochemical level and distinguish them from general stress-induced effects observed with *in vivo* rodent assays. Furthermore, cell assays allow for the use of RNA interferencemediated gene knockdowns, gene knockouts, or steroid precursors to selectively block or bypass certain reactions and isolate regions of the steroidogenic pathway for refinement of parameter estimates.

*Dynamic concentrationresponse behavior.* The model closely matched three dynamic concentrationresponse behaviors observed in these experiments. First, the concentration of the steroids (CORTICO, ALDO, CORT) downstream from CYP11B1 (enzyme inhibited by MET) decreased as MET increased (Figure 3AC). Second, the concentrations of steroids (DCORTICO and DCORT) immediately upstream of CYP11B1 slightly increased or remained constant as MET increased (Figure 3D,E). This small concentration increase in the model predictions and mean measurements is due to the decrease in the conversion rate of DCORTICO into CORTICO and of DCORT into CORT and the subsequent pooling of the substrates. Third, all the other steroids were unaffected by MET.

Our research goal is to better understand the doseresponse behavior of EACs. Our approach is to develop computational mechanistic models that describe the biological perturbations at the biochemical level and integrate information toward higher levels of biological organization. This approach will ultimately enable predictions of *in vivo* dose responses. To achieve this goal, further refinement of the model will be needed based on additional model-guided experiments, such as cell proliferation and viability, gene regulation, and upstream signaling.

*Limitations.* Although our model predictions compare well with the experimental data, the model-predicted concentrations of three steroids (PROG, PREG, and DCORTICO) do not correspond for a few measurements. For control experiments, the model underestimated PROG and PREG concentrations at 48 hr and overestimated them at 72 hr (Figure 2B,E), and underestimated DCORTICO concentrations at 8, 24, and 48 hr (Figure 2D). For MET experiments, DCORTICO did not correspond at 72 hr after incubation with 10 µM MET (Figure 3D). Instead of a small increase in DCORTICO as predicted by the model, MET had little or no effect on DCORTICO. Time-course measurements for these three steroids showed an increase in the mean concentrations until 48 hr, and then a sharp decrease (PROG and PREG) or no change (DCORTICO) at 72 hr. A possible source of these discrepancies is the model assumption of no saturation in the metabolic pathway; our model uses first-order enzyme kinetics. We plan to investigate a model with Michaelis-Menten enzyme kinetics that may improve the model fit.

## Conclusions

Our study demonstrates the ability of a newly developed mechanistic computational model of adrenal steroidogenesis to estimate the synthesis and secretion of adrenal steroids in human H295R cells, and their dynamic concentrationresponse to the EAC MET. Model-predicted steroid concentrations in the cells and medium closely correspond to the time-course measurements from control and MET-exposed cells. This capability could enhance the interpretation of data from *in vitro* steroidogenesis assays by helping to define mechanisms of action for poorly characterized chemicals and mixtures in support of *in vitro* EAC screening systems for predictive hazard assessments.

## Supplemental Material

(2.2 MB) PDF
