# Supplementary material for: Computational Model of Steroidogenesis in Human H295R Cells to Predict Biochemical Response to Endocrine-Active Chemicals: Model Development for Metyrapone
Source: Environ Health Perspect. 2009 Oct 16;118(2):265–72. doi: 10.1289/ehp.0901107 (PMC2831928; doi:10.1289/ehp.0901107)
Supplement: (2.2 MB) PDF [file ehp.0901107.s001.pdf]

Supplemental Material to:

Computational Model of Steroidogenesis in Human H295R Cells to Predict Biochemical Response to Endocrine Active Chemicals: Model Development for Metyrapone

Michael S. Breen,[1*] Miyuki Breen,[2,3] Natsuko Terasaki,[4] Makoto Yamazaki,[4] Rory B. Conolly[2]

[1] National Exposure Research Laboratory, U.S. Environmental Protection Agency, 109 T.W. Alexander Drive, Mail E205-02, Research Triangle Park, NC 27711, USA

[2] National Center for Computational Toxicology, U.S. Environmental Protection Agency, Research Triangle Park, NC 27711, USA

[3] Biomathematics Program, Department of Statistics, North Carolina State University, Raleigh, NC 27695, USA

[4] Safety Research Laboratory, Mitsubishi Tanabe Pharma Corporation, Kisarazu, Chiba 292-0818, Japan

Correspondence should be addressed to:
Michael S. Breen, Ph.D.
U.S. Environmental Protection Agency
Office of Research and Development
National Exposure Research Laboratory
109 T.W. Alexander Drive, Mail E205-02
Research Triangle Park, NC 27711
tel: 919-541-9409
fax: 919-541-9444
email: breen.michael@epa.gov

1. Steroidogenesis Assay with H295R cells

NCI H295R human adrenocortical carcinoma cells (American Type Culture Collection, Manassas, VA, USA) were grown in 1:1 mixture of Dulbecco's modified Eagle's medium (Invitrogen Corporation,Carlsbad, CA, USA) and Ham's F12 medium (MP Biomedicals Inc, Irvine, CA, USA) containing 15 mM HEPES (Dojindo Laboratories, Kumamoto, Japan), 0.00625 mg/ml insulin (Sigma-Aldrich, Inc., St. Louis, MO, USA), 0.00625 mg/ml transferrin (Sigma-Aldrich, Inc., St. Louis, MO, USA), 30 nmol/L sodium selenite (Wako Pure Chemical Industries, Ltd., Osaka, Japan), 1.25 mg/ml bovine serum albumin (Sigma-Aldrich, Inc., St. Louis, MO, USA), 0.00535 mg/ml linoleic acid (Sigma-Aldrich, Inc., St. Louis, MO, USA), 2.5 % Nu-Serum I (Becton, Dickinson and Company, Franklin Lakes, NJ , USA), 100 U/mL penicillin (Meiji Seika Kaisha, Ltd., Tokyo, Japan) and 100 mg/L streptomycin (Meiji Seika Kaisha Ltd., Tokyo, Japan) at 37°C in a 5% CO_2 atmosphere. Cells were grown in 225 cm^2 flask (Asahi Techno Glass Corporation, Chiba, Japan) to about 80 % confluence, and then split using 0.025% Trypsin (MP Biomedicals Inc, Irvine, California)- 0.02 % EDTA solution (Dojindo Laboratories, Kumamoto, Japan).

The control and MET exposure experiments were conducted in 6-well tissue culture plates (Becton, Dickinson and Company, Franklin Lakes, NJ, USA). A cell suspension of 2 ml containing $6x10^5$ cells was placed in each well. Each experiment had four replicates per time sample. The test plates were then incubated at 37°C in a 5% CO_2 atmosphere. After incubation for 72 hr, the medium was changed to either 2 ml of supplemented medium, which was1:1 mixture of Dulbecco's modified Eagle's medium and Ham's F12 medium (Invitrogen Corporation,Carlsbad, CA, USA) containing 0.00625 mg/ml insulin, 0.00625 mg/ml transferrin, 30 nmol/L sodium selenite, 1.25 mg/ml bovine serum albumin, 0.00535 mg/ml linoleic acid, 10 % FBS (Invitrogen Corporation, Carlsbad, CA, USA), 100 U/mL penicillin and 100 mg/L streptomycin, with 50 nM of adrenocorticotropin (ACTH, Sigma-Aldrich, Inc., St. Louis, MO, USA), 20 μM of forskolin (Sigma-Aldrich, Inc., St.

Louis, MO, USA), 100 nM of angiotensin II (EMD Chemicals Inc., Darmstadt, Germany) and 0.1% of dimethyl sulfoxide (DMSO,Wako Pure Chemical Industries, Ltd., Osaka, Japan), in the case of the control experiments, or 2 ml of MET-treated supplemented medium. Dilutions of a MET (Sigma-Aldrich, Inc., St. Louis, MO, USA) stock solution were prepared directly in supplemented medium to generate two test concentrations of MET (1 and 10 μM). After changing the medium, the test plates were incubated at 37°C with a 5% CO_2 atmosphere, and the experiments were initiated. At incubation periods of 8, 24, 48, and 72 hr for the control and MET experiments, the medium and cells were separately removed from four replicate wells. The cells were dissolved in 100 μl of distilled water and sonicated to produce a cell lysate.

The concentrations of twelve steroids (PREG, HPREG, DHEA, PROG, HPROG, DIONE, T, DCORTICO, CORTICO, ALDO, DCORT, CORT) in the medium and cell lysate were measured using liquid chromatography/mass spectrometry (LC-MS/MS). The LC-MS/MS systems consisted of a LC10A VP series (Shimadzu, Kyoto, Japan) and API4000 (Applied Biosystems, Foster City, CA, USA.). The steroids were extracted from the medium and cell lysate by ethyl acetate and separated on LC by acetonitrile and formic acid. MS/MS parameters were optimized using multiple reaction monitoring (MRM) mode for every steroids in positive electrospray ionization. The medium and cell lysate volumes were 500 μL and 70 μL, respectively, and LC-MS/MS running time was 17.5 min/sample. The concentrations of two additional steroids (E1, E2) in the medium and cell lysate were measured using enzyme-linked immunosorbent assay (ELISA) with commercial kits (Wako Pure Chemical Industries, Ltd., Osaka, Japan). The concentration of cholesterol in the medium and cell lysate was measured using a commercial kit (Wako Pure Chemical Industries, Ltd., Osaka, Japan) based on cholesterol oxidase method (Allain et al. 1974).

2. Dynamic molecular balance equations in cells and medium

CHOL in cells:

$$V_{\text{cell}}\frac{dC_{\text{CHOL,cell}}(t)}{dt}=k_1V_{\text{med}}C_{\text{CHOL,med}}(t)-k_2V_{\text{cell}}C_{\text{CHOL,cell}}(t);$$
$$C_{\text{CHOL,cell}}(0)=1.88\text{x}10^7\ \text{nM}$$

PREG in cells:

$$V_{\text{cell}}\frac{dC_{\text{PREG,cell}}(t)}{dt}=k_2V_{\text{cell}}C_{\text{CHOL,cell}}(t)-(k_3+k_5+k_{+19})V_{\text{cell}}C_{\text{PREG,cell}}(t)+k_{-19}V_{\text{med}}C_{\text{PREG,med}}(t);$$
$$C_{\text{PREG,cell}}(0)=9.45\text{x}10^3\ \text{nM}$$

HPREG in cells:

$$V_{\text{cell}}\frac{dC_{\text{HPREG,cell}}(t)}{dt}=k_3V_{\text{cell}}C_{\text{PREG,cell}}(t)-(k_4+k_6+k_{+24})V_{\text{cell}}C_{\text{HPREG,cell}}(t)+k_{-24}V_{\text{med}}C_{\text{HPREG,med}}(t);$$
$$C_{\text{HPREG,cell}}(0)=0$$

DHEA in cells:

$$V_{\text{cell}}\frac{dC_{\text{DHEA,cell}}(t)}{dt}=k_4V_{\text{cell}}C_{\text{HPREG,cell}}(t)-(k_7+k_{+28})V_{\text{cell}}C_{\text{DHEA,cell}}(t)+k_{-28}V_{\text{med}}C_{\text{DHEA,med}}(t);$$
$$C_{\text{DHEA,cell}}(0)=3.49\text{x}10^3\ \text{nM}$$

PROG in cells:

$$V_{\text{cell}}\frac{dC_{\text{PROG,cell}}(t)}{dt}=k_5V_{\text{cell}}C_{\text{PREG,cell}}(t)-(k_8+k_{11}+k_{+20})V_{\text{cell}}C_{\text{PROG,cell}}(t)+k_{\text{-}20}V_{\text{med}}C_{\text{PROG,med}}(t);$$
$$C_{\text{PROG,cell}}(0)=32.10\ \text{nM}$$

HPROG in cells:

$$V_{\text{cell}}\frac{dC_{\text{HPROG,cell}}(t)}{dt}=k_6V_{\text{cell}}C_{\text{HPREG,cell}}(t)+k_8V_{\text{cell}}C_{\text{PROG,cell}}(t)-(k_9+k_{12}+k_{+25})V_{\text{cell}}C_{\text{HPROG,cell}}(t)+k_{-25}V_{\text{med}}C_{\text{HPROG,med}}(t);$$
$$C_{\text{HPROG,cell}}(0)=101.83\ \text{nM}$$

DIONE in cells:

$$V_{\text{cell}}\frac{dC_{\text{DIONE,cell}}(t)}{dt}=k_7V_{\text{cell}}C_{\text{DHEA,cell}}(t)+k_9V_{\text{cell}}C_{\text{HPROG,cell}}(t)-(k_{10}+k_{13}+k_{+29})V_{\text{cell}}C_{\text{DIONE,cell}}(t)+k_{-29}V_{\text{med}}C_{\text{DIONE,med}}(t);$$
$$C_{\text{DIONE,cell}}(0)=2.33\text{x}10^3\ \text{nM}$$

T in cells:

$$V_{\text{cell}}\frac{dC_{\text{T,cell}}(t)}{dt}=k_{10}V_{\text{cell}}C_{\text{AD,cell}}(t)-(k_{14}+k_{+31})V_{\text{cell}}C_{\text{T,cell}}(t)+k_{-31}V_{\text{med}}C_{\text{T,med}}(t)$$
$$C_{\text{T,cell}}(0)=0$$

E1 in cells:

$$V_{\text{cell}}\frac{dC_{\text{E1,cell}}(t)}{dt}=k_{13}V_{\text{cell}}C_{\text{DIONE,cell}}(t)-(k_{15}+k_{+30})V_{\text{cell}}C_{\text{E1,cell}}(t)+k_{-30}V_{\text{med}}C_{\text{E1,med}}(t)$$
$$C_{\text{E1,cell}}(0)=2.09\text{x}10^3\ \text{nM}$$

E2 in cells:

$$V_{\text{cell}}\frac{dC_{\text{E2,ovy}}(t)}{dt}=k_{14}V_{\text{cell}}C_{\text{T,cell}}(t)+k_{15}V_{\text{cell}}C_{\text{E1,cell}}(t)-k_{+32}V_{\text{cell}}C_{\text{E2,cell}}(t)+k_{-32}V_{\text{med}}C_{\text{E2,med}}(t)$$
$$C_{\text{E2,cell}}(0)=424.17\ \text{nM}$$

DCORTICO in cells:

$$V_{\text{cell}}\frac{dC_{\text{DCORTICO,cell}}(t)}{dt}=k_{11}V_{\text{cell}}C_{\text{PROG,cell}}(t)-\left(\frac{k_{16}}{\alpha_{\text{CORTICO}}}+k_{+21}\right)V_{\text{cell}}C_{\text{DCORTICO,cell}}(t)+k_{-21}V_{\text{med}}C_{\text{DCORTICO,med}}(t)$$
$$C_{\text{DCORTICO,cell}}(0)=835.01\ \text{nM}$$

CORTICO in cells:

$$V_{\text{cell}}\frac{dC_{\text{CORTICO,cell}}(t)}{dt}=\frac{k_{16}}{\alpha_{\text{CORTICO}}}V_{\text{cell}}C_{\text{DCORTICO,cell}}(t)-(k_{18}+k_{+22})V_{\text{cell}}C_{\text{CORTICO,cell}}(t)+k_{-22}V_{\text{med}}C_{\text{CORTICO,med}}(t)$$
$$C_{\text{CORTICO,cell}}(0)=2.27\text{x}10^3\ \text{nM}$$

ALDO in cells:

$$V_{\text{cell}} \frac{dC_{\text{ALDO,cell}}(t)}{dt} = k_{18} V_{\text{cell}} C_{\text{CORTICO,cell}}(t) - k_{+23} V_{\text{cell}} C_{\text{ALDO,cell}}(t) + k_{-23} V_{\text{med}} C_{\text{ALDO,med}}(t)$$

$$C_{\text{ALDO,cell}}(0) = 0$$

DCORT in cells:

$$V_{\text{cell}} \frac{dC_{\text{DCORT,cell}}(t)}{dt} = k_{12} V_{\text{cell}} C_{\text{HPROG,cell}}(t) - \left(\frac{k_{17}}{\alpha_{\text{CORT}}} + k_{+26} \right) V_{\text{cell}} C_{\text{DCORT,cell}}(t) + k_{-26} V_{\text{med}} C_{\text{DCORT,med}}(t)$$

$$C_{\text{DCORT,cell}}(0) = 7.56\text{x}10^4 \text{ nM}$$

CORT in cells:

$$V_{\text{cell}} \frac{dC_{\text{CORT,cell}}(t)}{dt} = \frac{k_{17}}{\alpha_{\text{CORT}}} V_{\text{cell}} C_{\text{DCORT,cell}}(t) - k_{+27} V_{\text{cell}} C_{\text{CORT,cell}}(t) + k_{-27} V_{\text{med}} C_{\text{CORT,med}}(t)$$

$$C_{\text{CORT,cell}}(0) = 3.43\text{x}10^3 \text{ nM}$$

CHOL in medium:

$$\frac{dC_{\text{CHOL,med}}(t)}{dt} = -k_1 C_{\text{CHOL,med}}(t);\ C_{\text{CHOL,med}}(0) = 8.11\text{x}10^4 \text{ nM}$$

PREG in medium:

$$V_{\text{med}} \frac{dC_{\text{PREG,med}}(t)}{dt} = k_{+19} V_{\text{cell}} C_{\text{PREG,cell}}(t) - k_{-19} V_{\text{med}} C_{\text{PREG,med}}(t);\ C_{\text{PREG,med}}(0) = 0.85 \text{ nM}$$

HPREG in medium:

$$V_{\text{med}} \frac{dC_{\text{HPREG,med}}(t)}{dt} = k_{+24} V_{\text{cell}} C_{\text{HPREG,cell}}(t) - k_{-24} V_{\text{med}} C_{\text{HPREG,med}}(t);\ C_{\text{HPREG,med}}(0) = 69.45 \text{ nM}$$

DHEA in medium:

$$V_{\text{med}} \frac{dC_{\text{DHEA,med}}(t)}{dt} = k_{+28} V_{\text{cell}} C_{\text{DHEA,cell}}(t) - k_{-28} V_{\text{med}} C_{\text{DHEA,med}}(t);\ C_{\text{DHEA,med}}(0) = 0$$

PROG in medium:

$$V_{\mathrm{med}}\frac{dC_{\mathrm{PROG,med}}(t)}{dt}=k_{+20}V_{\mathrm{cell}}C_{\mathrm{PROG,cell}}(t)-k_{-20}V_{\mathrm{med}}C_{\mathrm{PROG,med}}(t);\ C_{\mathrm{PROG,med}}(0)=0.03\ \mathrm{nM}$$

HPROG in medium:

$$V_{\mathrm{med}}\frac{dC_{\mathrm{HPROG,med}}(t)}{dt}=k_{+25}V_{\mathrm{cell}}C_{\mathrm{HPROG,cell}}(t)-k_{-25}V_{\mathrm{med}}C_{\mathrm{HPROG,med}}(t);\ C_{\mathrm{HPROG,med}}(0)=0$$

DIONE in medium:

$$V_{\mathrm{med}}\frac{dC_{\mathrm{DIONE,med}}(t)}{dt}=k_{+29}V_{\mathrm{cell}}C_{\mathrm{DIONE,cell}}(t)-k_{-29}V_{\mathrm{med}}C_{\mathrm{DIONE,med}}(t);\ C_{\mathrm{DIONE,med}}(0)=0.80\ \mathrm{nM}$$

T in medium:

$$V_{\mathrm{med}}\frac{dC_{\mathrm{T,med}}(t)}{dt}=k_{+31}V_{\mathrm{cell}}C_{\mathrm{T,cell}}(t)-k_{-31}V_{\mathrm{med}}C_{\mathrm{T,med}}(t);\ C_{\mathrm{T,med}}(0)=0.80\ \mathrm{nM}$$

E1 in medium:

$$V_{\mathrm{med}}\frac{dC_{\mathrm{E1,med}}(t)}{dt}=k_{+30}V_{\mathrm{cell}}C_{\mathrm{E1,cell}}(t)-k_{-30}V_{\mathrm{med}}C_{\mathrm{E1,med}}(t);\ C_{\mathrm{E1,med}}(0)=0.11\ \mathrm{nM}$$

E2 in medium:

$$V_{\mathrm{med}}\frac{dC_{\mathrm{E2,med}}(t)}{dt}=k_{+32}V_{\mathrm{cell}}C_{\mathrm{E2,cell}}(t)-k_{-32}V_{\mathrm{med}}C_{\mathrm{E2,med}}(t);\ C_{\mathrm{E2,med}}(0)=1.21\ \mathrm{nM}$$

DCORTICO in medium:

$$V_{\mathrm{med}}\frac{dC_{\mathrm{DCORTICO,med}}(t)}{dt}=k_{+21}V_{\mathrm{cell}}C_{\mathrm{DCORTICO,cell}}(t)-k_{-21}V_{\mathrm{med}}C_{\mathrm{DCORTICO,med}}(t);\ C_{\mathrm{DCORTICO,med}}(0)=0\ \mathrm{nM}$$

CORTICO in medium:

$$V_{\mathrm{med}}\frac{dC_{\mathrm{CORTICO,med}}(t)}{dt}=k_{+22}V_{\mathrm{cell}}C_{\mathrm{CORTICO,cell}}(t)-k_{-22}V_{\mathrm{med}}C_{\mathrm{CORTICO,med}}(t);\ C_{\mathrm{CORTICO,med}}(0)=0.11\ \mathrm{nM}$$

ALDO in medium:

$$V_{\text{med}} \frac{dC_{\text{ALDO,med}}(t)}{dt} = k_{+23} V_{\text{cell}} C_{\text{ALDO,cell}}(t) - k_{-23} V_{\text{med}} C_{\text{ALDO,med}}(t);\; C_{\text{ALDO,med}}(0) = 0.91\ \text{nM}$$

DCORT in medium:

$$V_{\text{med}} \frac{dC_{\text{DCORT,med}}(t)}{dt} = k_{+26} V_{\text{cell}} C_{\text{DCORT,cell}}(t) - k_{-26} V_{\text{med}} C_{\text{DCORT,med}}(t);\; C_{\text{DCORT,med}}(0) = 0\ \text{nM}$$

CORT in medium:

$$V_{\text{med}} \frac{dC_{\text{CORT,med}}(t)}{dt} = k_{+27} V_{\text{cell}} C_{\text{CORT,cell}}(t) - k_{-27} V_{\text{med}} C_{\text{CORT,med}}(t);\; C_{\text{CORT,med}}(0) = 0.03\ \text{nM}$$

3. Molecular balance equations for quasi-equilibrium

CHOL in cells:

$$V_{\text{cell}} \frac{dC_{\text{CHOL,cell}}(t)}{dt} = k_1 V_{\text{med}} C_{\text{CHOL,med}}(t) - k_2 V_{\text{cell}} C_{\text{CHOL,cell}}(t);\; C_{\text{CHOL,cell}}(0) = 1.88\text{x}10^7\ \text{nM}$$

PREG in cells:

$$\frac{dC_{\text{PREG,cell}}(t)}{dt} = \left(\frac{1}{V_{\text{cell}} + V_{\text{med}} q_{19}}\right)\left[k_2 V_{\text{cell}} C_{\text{CHOL,cell}}(t) - (k_3 + k_5) V_{\text{cell}} C_{\text{PREG,cell}}(t)\right];\; C_{\text{PREG,cell}}(0) = 9.45\text{x}10^3\ \text{nM}$$

HPREG in cells:

$$\frac{dC_{\text{HPREG,cell}}(t)}{dt} = \left(\frac{1}{V_{\text{cell}} + V_{\text{med}} q_{24}}\right)\left[k_3 V_{\text{cell}} C_{\text{PREG,cell}}(t) - (k_4 + k_6) V_{\text{cell}} C_{\text{HPREG,cell}}(t)\right];\; C_{\text{HPREG,cell}}(0) = 0$$

DHEA in cells:

$$\frac{dC_{\text{DHEA,cell}}(t)}{dt} = \left(\frac{1}{V_{\text{cell}} + V_{\text{med}} q_{28}}\right)\left[k_4 V_{\text{cell}} C_{\text{HPREG,cell}}(t) - k_7 V_{\text{cell}} C_{\text{DHEA,cell}}(t)\right];\; C_{\text{DHEA,cell}}(0) = 3.49\text{x}10^3\ \text{nM}$$

PROG in cells:

$$\frac{dC_{\mathrm{PROG,cell}}(t)}{dt}=\left(\frac{1}{V_{\mathrm{cell}}+V_{\mathrm{med}}q_{20}}\right)\left[k_5V_{\mathrm{cell}}C_{\mathrm{PREG,cell}}(t)-(k_8+k_{11})V_{\mathrm{cell}}C_{\mathrm{PROG,cell}}(t)\right];\ C_{\mathrm{PROG,cell}}(0)=32.10\ \mathrm{nM}$$

HPROG in cells:

$$\frac{dC_{\mathrm{HPROG,cell}}(t)}{dt}=\left(\frac{1}{V_{\mathrm{cell}}+V_{\mathrm{med}}q_{25}}\right)\left[k_6V_{\mathrm{cell}}C_{\mathrm{HPREG,cell}}(t)+k_8V_{\mathrm{cell}}C_{\mathrm{PROG,cell}}(t)-(k_9+k_{12})V_{\mathrm{cell}}C_{\mathrm{HPROG,cell}}(t)\right]$$

$$C_{\mathrm{HPROG,cell}}(0)=101.83\ \mathrm{nM}$$

DIONE in cells:

$$\frac{dC_{\mathrm{DIONE,cell}}(t)}{dt}=\left(\frac{1}{V_{\mathrm{cell}}+V_{\mathrm{med}}q_{29}}\right)\left[k_7V_{\mathrm{cell}}C_{\mathrm{DHEA,cell}}(t)+k_9V_{\mathrm{cell}}C_{\mathrm{HPROG,cell}}(t)-(k_{10}+k_{13})V_{\mathrm{cell}}C_{\mathrm{DIONE,cell}}(t)\right]$$

$$C_{\mathrm{DIONE,cell}}(0)=2.33\mathrm{x}10^3\ \mathrm{nM}$$

T in cells:

$$\frac{dC_{\mathrm{T,cell}}(t)}{dt}=\left(\frac{1}{V_{\mathrm{cell}}+V_{\mathrm{med}}q_{31}}\right)\left[k_{10}V_{\mathrm{cell}}C_{\mathrm{DIONE,cell}}(t)-k_{14}V_{\mathrm{cell}}C_{\mathrm{T,cell}}(t)\right];\ C_{\mathrm{T,cell}}(0)=0$$

E1 in cells:

$$\frac{dC_{\mathrm{E1,cell}}(t)}{dt}=\left(\frac{1}{V_{\mathrm{cell}}+V_{\mathrm{med}}q_{30}}\right)\left[k_{13}V_{\mathrm{cell}}C_{\mathrm{DIONE,cell}}(t)-k_{15}V_{\mathrm{cell}}C_{\mathrm{E1,cell}}(t)\right];\ C_{\mathrm{E1,cell}}(0)=2.09\mathrm{x}10^3\ \mathrm{nM}$$

E2 in cells:

$$\frac{dC_{\mathrm{E2,ovy}}(t)}{dt}=\left(\frac{1}{V_{\mathrm{cell}}+V_{\mathrm{med}}q_{32}}\right)\left[k_{14}V_{\mathrm{cell}}C_{\mathrm{T,cell}}(t)+k_{15}V_{\mathrm{cell}}C_{\mathrm{E1,cell}}(t)\right];\ C_{\mathrm{E2,cell}}(0)=424.17\ \mathrm{nM}$$

DCORTICO in cells:

$$\frac{dC_{\text{DCORTICO,cell}}(t)}{dt}=\left(\frac{1}{V_{\text{cell}}+V_{\text{med}}q_{21}}\right)\left[k_{11}V_{\text{cell}}C_{\text{PROG,cell}}(t)-\left(\frac{k_{16}}{\alpha_{\text{CORTICO}}}\right)V_{\text{cell}}C_{\text{DCORTICO,cell}}(t)\right];\ C_{\text{DCORTICO,cell}}(0)=835.01\,\text{nM}$$

CORTICO in cells:

$$\frac{dC_{\text{CORTICO,cell}}(t)}{dt}=\left(\frac{1}{V_{\text{cell}}+V_{\text{med}}q_{22}}\right)\left[\frac{k_{16}}{\alpha_{\text{CORTICO}}}V_{\text{cell}}C_{\text{DCORTICO,cell}}(t)-k_{18}V_{\text{cell}}C_{\text{CORTICO,cell}}(t)\right];\quad C_{\text{CORTICO,cell}}(0)=2.27\text{x}10^{3}\ \text{nM}$$

ALDO in cells:

$$\frac{dC_{\text{ALDO,cell}}(t)}{dt}=\left(\frac{1}{V_{\text{cell}}+V_{\text{med}}q_{23}}\right)\left[k_{18}V_{\text{cell}}C_{\text{CORTICO,cell}}(t)\right];\ C_{\text{ALDO,cell}}(0)=0$$

DCORT in cells:

$$\frac{dC_{\text{DCORT,cell}}(t)}{dt}=\left(\frac{1}{V_{\text{cell}}+V_{\text{med}}q_{26}}\right)\left[k_{12}V_{\text{cell}}C_{\text{HPROG,cell}}(t)-\left(\frac{k_{17}}{\alpha_{\text{CORT}}}\right)V_{\text{cell}}C_{\text{DCORT,cell}}(t)\right];\ C_{\text{DCORT,cell}}(0)=7.56\text{x}10^{4}\ \text{nM}$$

CORT in cells:

$$\frac{dC_{\text{CORT,cell}}(t)}{dt}=\left(\frac{1}{V_{\text{cell}}+V_{\text{med}}q_{27}}\right)\left[\frac{k_{17}}{\alpha_{\text{CORT}}}V_{\text{cell}}C_{\text{DCORT,cell}}(t)\right];\ C_{\text{CORT,cell}}(0)=3.43\text{x}10^{3}\ \text{nM}$$

FIGURE LEGENDS

Supplemental Material, Figure 1. Graphical representation of the parameters for the mathematical H295R steroidogenesis model. First-order rate constant for cholesterol uptake into the cells is k_1. First-order rate constants for metabolic processes are: $k_2 - k_{18}$. Reversible first-order rate constants for transport processes (k_{+x} and k_{-x} for secretion and import of steroid *x*; respectively) are

$k_{19} - k_{32}$. Enzyme inhibition constants for MET are k_{41} and k_{42} for CORTICO and CORT pathways, respectively.

Supplemental Material, Figure 2. Comparison of transport equilibrium model-predictions (linear regression line) with measurements in cells and medium. Model-predicted DCORTICO concentrations in medium were plotted as a function of DCORTICO concentrations in cells, and compared with mean concentrations measured at five sampling times for control and two MET concentrations.

Supplemental Material, Figure 3. Model evaluation of transport pathway. Comparison of transport equilibrium model-predictions with time-course measurements in medium from control (a) and two MET concentrations: 1 μM (b) and 10 μM (c). Model-predicted and mean measured DCORTICO concentrations in medium were plotted at five time points after incubation of cells with MET. Model-predicted DCORTICO concentrations in medium were estimated from mean measured concentrations in cells at each corresponding time point. Dotted lines represent linear interpolations between model-predicted and measured concentrations. Measured steroid concentrations are same as shown in Fig. SF2.

Supplemental Material, Figure 4. Model evaluation of metabolic pathway for control experiments. Model-predicted concentrations in cells were plotted as a function of time, and compared with concentrations (mean and standard deviation) measured at five sampling times for steroids: ALDO, E2, T (a); PROG, HPROG, DHEA (b); HPREG, DIONE, E1 (c); CORTICO, DCORTICO (d); PREG, CORT, DCORT (e).

Supplemental Material, Figure 5. Model evaluation of metabolic pathway for control and MET-exposed cells. Model-predicted concentrations in cells were plotted as a function of time, and compared with concentrations (mean and standard deviation) measured at five sampling times for steroids: ALDO (a), CORTICO (b), CORT (c), DCORTICO (d), DCORT (e). For controls, model-predicted and measured steroid concentrations are same as shown in Fig. SF4.

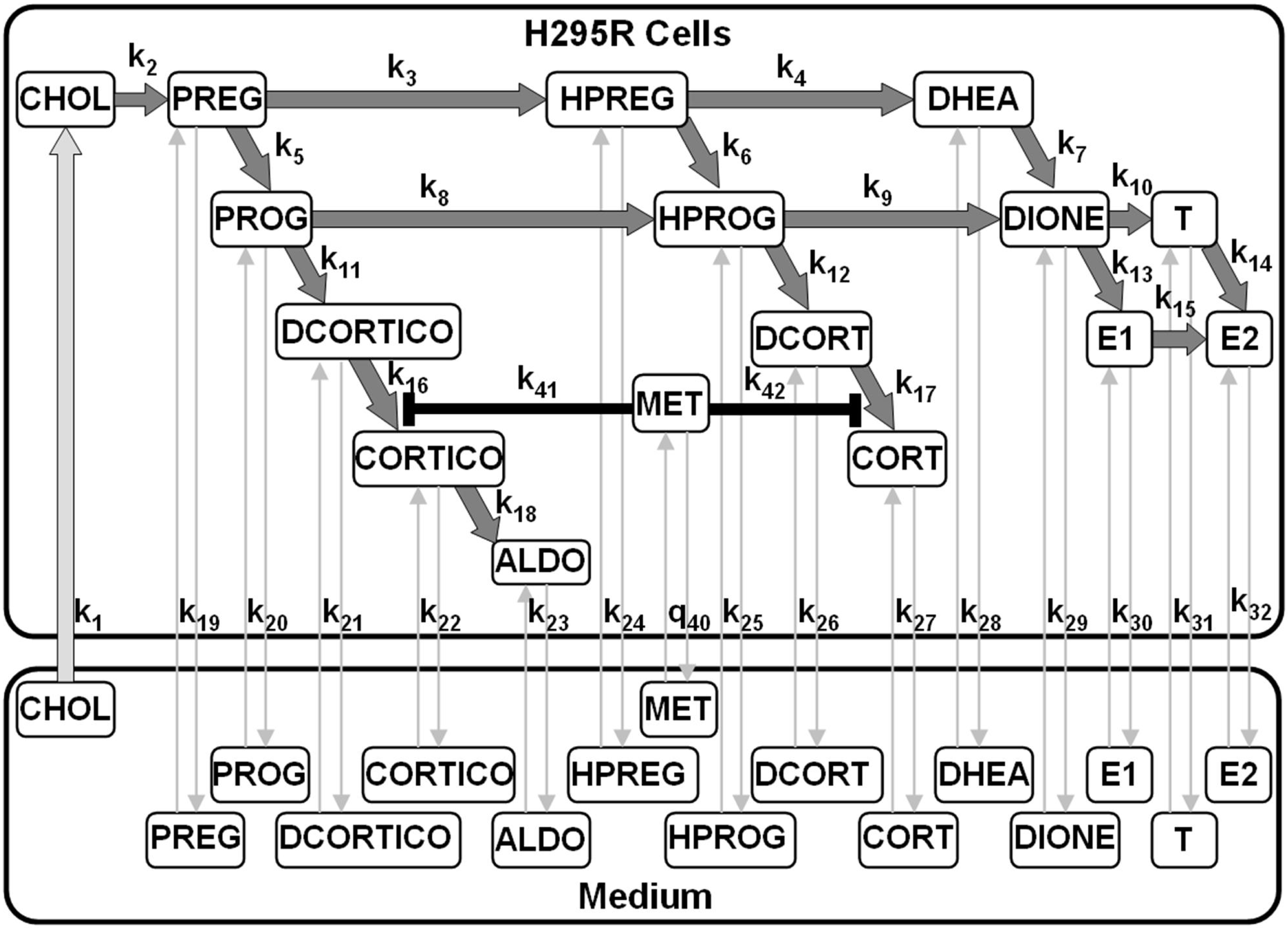

Supplemental Material, Figure 1

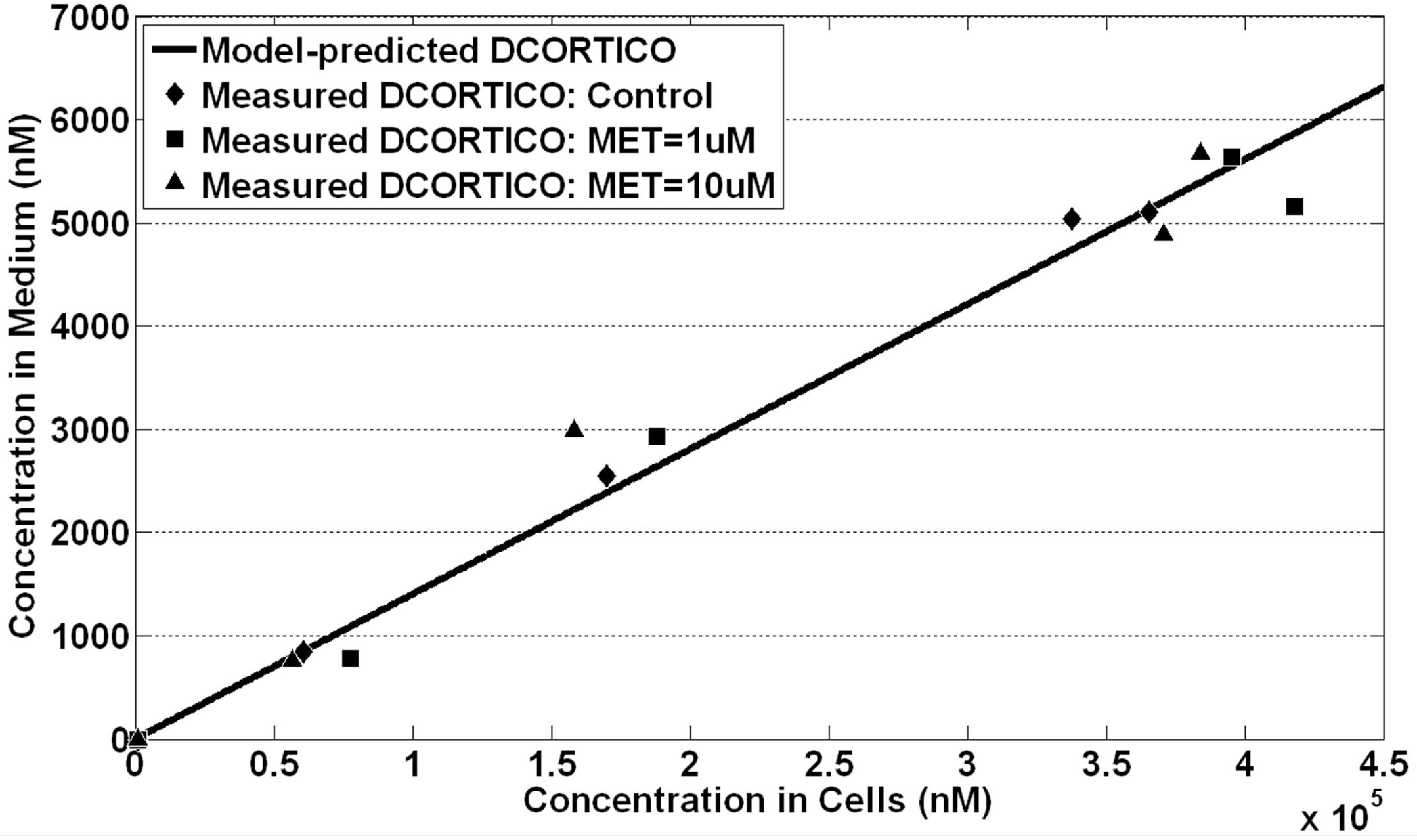

Supplemental Material, Figure 2

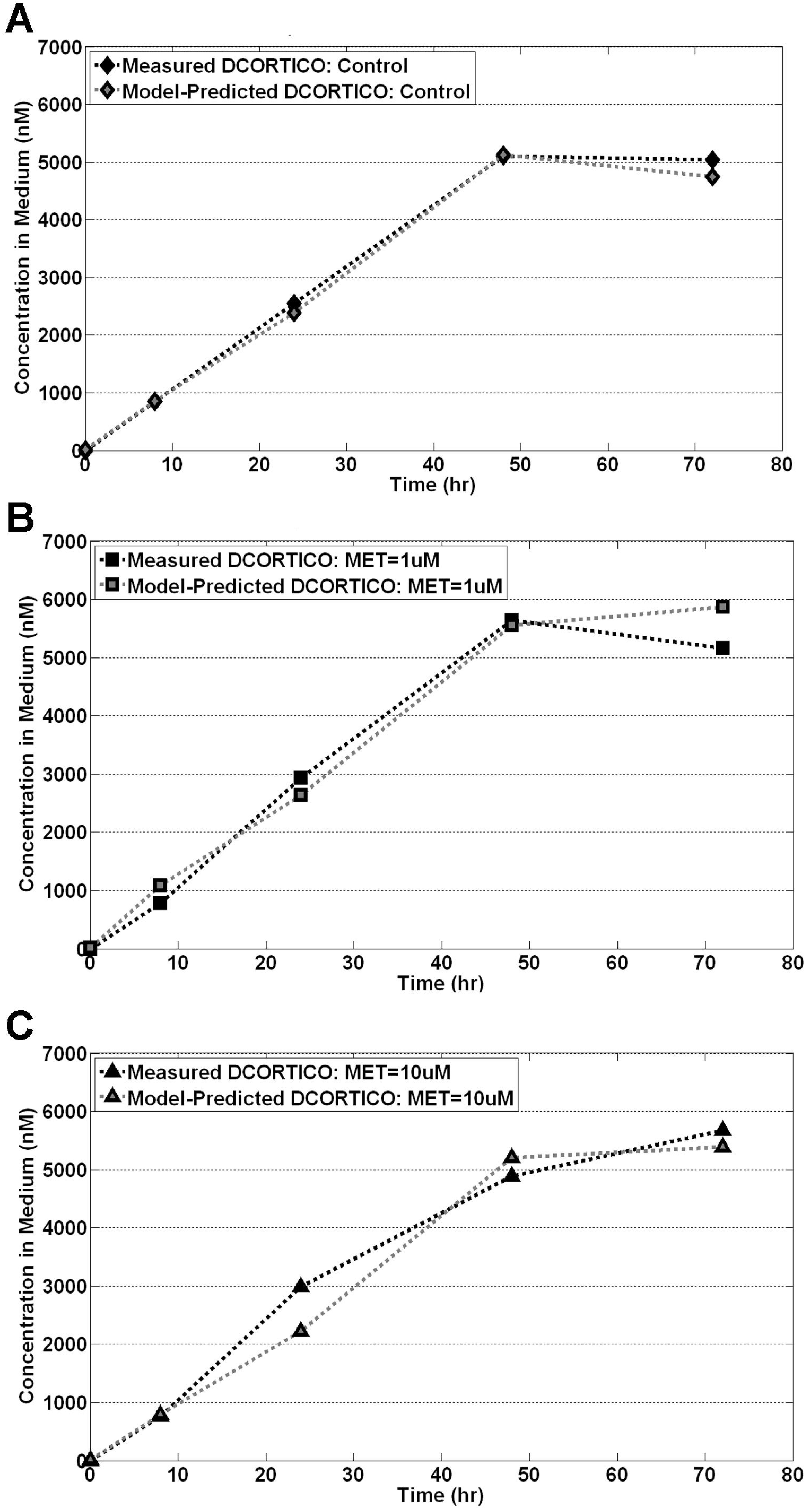

Supplemental Material, Figure 3

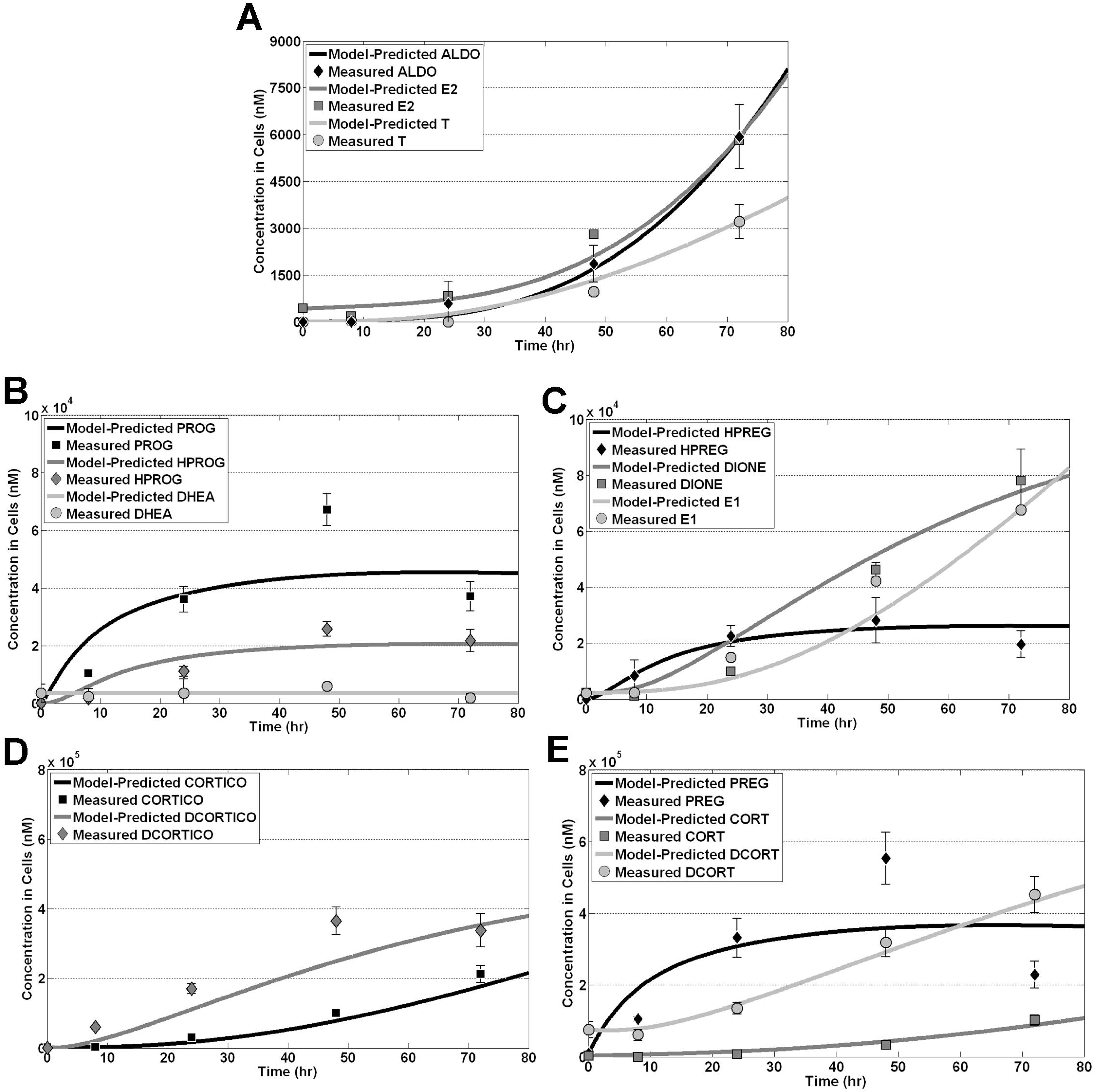

Supplemental Material, Figure 4

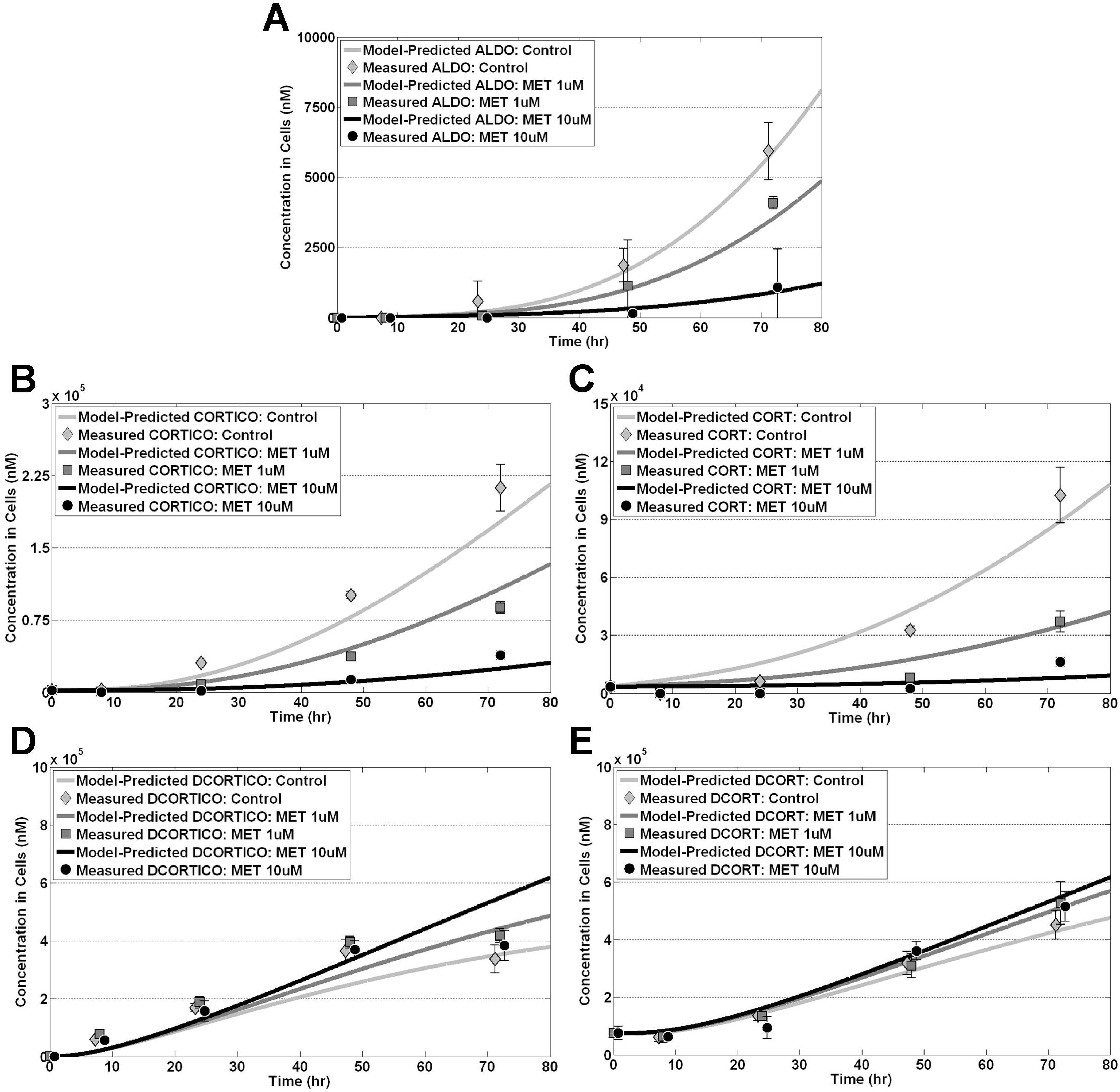

Supplemental Material, Figure 5

Supplemental Material, Table 1. Quantitative ranges for steroids in cells and medium

Steroid	Quantitative range (nM) Cells	Medium
PREG	$1.3x10^4 – 1.3x10^6$	$15.8 – 1.6x10^3$
HPREG	$1.2x10^4 – 1.2x10^6$	$15.0 – 1.5x10^3$
DHEA	$1.4x10^4 – 1.4x10^6$	$17.3 – 1.7x10^3$
PROG	$2.6x10^3 – 1.3x10^6$	$3.2 – 1.6x10^3$
HPROG	$2.4x10^3 – 1.2x10^6$	$3.0 – 1.5x10^3$
DIONE	$2.8x10^3 – 1.4x10^6$	$3.5 – 1.7x10^3$
T	$2.8x10^3 – 1.4x10^6$	$3.5 – 1.7x10^3$
E1	$1.6x10^3 – 1.5x10^5$	$5.5 – 5.5x10^2$
E2	$5.0x10^2 – 1.0x10^4$	$5.5 – 1.1x10^2$
DCORTICO	$1.2x10^4 – 6.1x10^6$	$15.1 – 7.6x10^3$
CORTICO	$1.2x10^4 – 5.9x10^6$	$14.4 – 7.2x10^3$
ALDO	$2.2x10^3 – 1.1x10^6$	$2.8 – 1.4x10^3$
DCORT	$1.2x10^4 – 5.9x10^6$	$14.4 – 7.2x10^3$
CORT	$1.1x10^4 – 5.6x10^6$	$13.8 – 6.9x10^3$

Supplemental Material, Table 1
